# In Vivo Wound Healing Effects of Antimicrobial Peptide‐Based Dressings for *S. aureus*‐Infected Wounds—A Systematic Review and Meta‐Analysis

**DOI:** 10.1111/wrr.70183

**Published:** 2026-06-22

**Authors:** Lea Rodge, Artemis Stamboulis, Sarah A. Kuehne, Melissa M. Grant, Zubair Ahmed

**Affiliations:** ^1^ Biomaterials Research Group, School of Metallurgy and Materials, College of Engineering and Physical Sciences, University of Birmingham Birmingham UK; ^2^ Nottingham Trent University, School of Science and Technology Nottingham UK; ^3^ Periodontal Research Group, Dentistry, School of Health Sciences, College of Medicine and Health, University of Birmingham Birmingham UK; ^4^ Birmingham Community Health NHS Foundation Trust Birmingham Dental Hospital Birmingham UK; ^5^ NIHR Birmingham Biomedical Research Centre, University of Birmingham Birmingham UK; ^6^ Neuroscience and Ophthalmology, Department of Inflammation and Ageing School of Infection, Inflammation and Immunology, University of Birmingham Birmingham UK; ^7^ Centre for Trauma Sciences Research, University of Birmingham Birmingham UK; ^8^ University Hospitals Birmingham NHS Trust Birmingham UK

**Keywords:** antimicrobial peptides, dressing, in vivo, infection, wound healing

## Abstract

The purpose of this systematic review was to determine whether the addition of antimicrobial peptides (AMPs) to dressing materials enhances their wound healing capabilities when applied to *S. aureus* ‐infected wounds in rodent models. The primary objective was not to derive definitive efficacy conclusions, but to systematically assess the quality, consistency, and translational readiness of existing in vivo evidence for AMP‐based dressings in excisional wound models to inform future in vivo experimental design. The systematic review was performed according to Preferred Reporting Items for Systematic Reviews and Meta‐Analyses guidelines. Two authors independently completed the literature search using three electronic databases and two databases for grey literature. Studies were included if they met pre‐established inclusion criteria. Rate of wound healing was considered as the primary outcome; the secondary outcome involved histological analyses of tissue samples of the wounds. Risk of bias was assessed using the SYRCLE risk of bias tool for animal studies and meta‐analysis was performed with pooled data from three or more studies reporting the same outcome, employing a random effects model. By the final timepoint, the meta‐analyses identified a significant increase in wound healing rate in both rats (mean difference = 2.79%, 95% CI [1.52%, 4.06%], *p* < 0.0001) and mice (mean difference = 32.30%, 95% CI [18.06%, 46.54%], *p* < 0.00001) exposed to AMP‐based dressing materials compared to AMP‐free carrier materials. The significantly improved wound healing rates provide promising evidence that these materials are suitable for promoting the wound healing of *S. aureus* ‐infected wounds that may be investigated further in clinical studies.

## 1 Introduction

Antimicrobial resistance (AMR) is an incessant issue across the clinical setting. Over‐prescription and a lack of novel antibiotics development are contributing to a continuous decline in the efficacy of conventional antibiotics. According to a study commissioned by the UK government, it is estimated that AMR will be responsible for 10 million deaths annually by 2050 [1]. In burn wounds, infection, particularly by multidrug resistant pathogens, is a prevalent complication. Although burn wounds are initially sterile, infection can occur through re‐colonisation by the patient's own microbiome or via nosocomial transmission [2, 3]. Infection may interfere with the healing process, increasing the hospital stay duration and, more critically, reducing the probability of survival of the patient [4, 5].

In the context of AMR, several pathogens have been recognised as being of special interest due to their high rates of resistance and high prevalence in the clinical setting, termed ‘ESKAPE’ pathogens. These are: *Enterococcus faecium, Staphylococcus aureus, Klebsiella pneumoniae, Acinetobacter baumannii, Pseudomonas aeruginosa* and *Enterobacter* spp. [6]. Due to its presence in the skin microbiota, *S. aureus* is one of the most common pathogens associated with the colonisation of burn wounds and becomes pathogenic when it begins to penetrate into deeper tissues of the wound bed [7].

As a result of AMR, the search for alternatives to antibiotics is becoming a priority with increasing importance. Preferably, the alternative should maintain comparable antimicrobial potency whilst demonstrating reduced levels of resistance compared to conventional antibiotic treatment [8]. Antimicrobial peptides (AMPs) are attractive therapeutic candidates because, unlike antibiotics, they do not target a single molecule and instead exhibit broad‐spectrum activity. This arises from their cationic charge, which enables electrostatic interactions with the anionic bacterial membrane, ultimately leading to bacterial cell lysis. In addition, AMPs can interfere with DNA replication and transcription, as well as protein and cell wall synthesis [9, 10, 11].

Besides their direct antimicrobial activity, a number of AMPs have known functions in immunomodulation, a further property that makes them attractive for wound healing purposes [12]. For example, LL‐37, an AMP derived from the protein cathelicidin, exhibits both pro‐ and anti‐inflammatory effects. Pro‐inflammatory roles include the negative regulation of IL‐10 and the positive regulation of IL‐8, IL‐12p40, and IL‐1β [13], whilst anti‐inflammatory functions include the inhibition of the Absent in melanoma 2 (AIM2) inflammasome and the suppression of IFN‐γ, TNF‐α, IL‐4 and IL‐12 [14]. For this reason, using AMP‐based formulations may provide materials that can accelerate recovery from wounds, not only by reducing the pathogenic load, but also actively encouraging the healing process via immune regulation.

Despite an abundance of research pertaining to AMPs, there is a lack of AMP‐based therapeutics currently available commercially. The aim of this systematic review was to evaluate the evidence base for AMP‐based dressing materials as candidates for wound healing, and to assess their effects in rodent‐based animal models. This body of work was not intended to establish definitive efficacy of AMP‐based dressings, but rather to inform future in vivo investigations by systematically assessing study design, consistency and translational readiness.

## 2 Methods

### 2.1 Literature Search

The Preferred Reporting Items for Systematic Reviews and Meta‐Analyses (PRISMA) guidelines were consulted in the development of the research strategy, with modifications to account for the animal studies [15]. Three databases were used in the literature search: EMBASE, Web of Science, and MEDLINE. Ethos and Cochrane Library were searched for grey literature. Two independent reviewers (LW and ZA) performed the search using the following identical search terms across all of the databases: (antimicrobial peptides OR AMP) AND (Collagen OR alginate OR cellulose OR silk fibroin OR PEG or PLA OR polyethylene glycol OR polycaprolactone) AND (cutaneous OR dermal OR wound OR infection) (see Table S1 for full search operators). Searches were restricted to English language and published during or after 2013 until February 2025. Duplicates were removed, then titles and abstracts were compared to the eligibility criteria which had been established in advance. Once filtered according to titles and abstracts, the full texts of all remaining included studies were assessed against the eligibility criteria; only studies that met all inclusion criteria were included in the review.

### 2.2 Inclusion and Exclusion Criteria

Inclusion and exclusion criteria were established and divided according to animal, intervention, control, and study type characteristics (PICOS) of the candidate studies (Table 1).

**TABLE 1:** Inclusion and exclusion criteria for studies to be included in the systematic review.

	Inclusion criteria	Exclusion criteria
Animal	Male and/or female mice or rats (any strain) Induced cutaneous excision wounds that have been infected with *S. aureus*	Any other animal species Animals with any other morbidities Infected with any other pathogen but not *S. aureus*
Intervention	Topical application of a dressing containing any AMP May include hydrogels or nanofiber‐based dressings	Systemic delivery therapeutics Dressings containing other major active therapeutics (e.g., antibiotics) Topical creams, ointments or sprays
Control	Topical application of dressing containing no AMPs. May be the material used in the intervention group with no AMPs included, or another dressing which is widely used for cutaneous wounds and does not contain AMPs.	No‐treatment control groups as the only control group
Study type	In vivo infected excision wound model	Studies containing no in vivo model, studies with no control group

*Note:* Criteria were divided into four groups: animal, intervention, control, and study type, following the frequently used PICOS structure.

### 2.3 Data Collection

Data collection forms and a data collection table were used to collect data from all included studies. Forms were completed for all studies individually, which were then used to complete a data extraction table; forms were piloted against two studies and amended as needed. Subsequently, the format of forms was kept identical for all further studies. Data to be extracted included study and subject characteristics and outcome data. Data collection was completed independently by two reviewers (LW and ZA), and any disagreements were resolved through discussion. To reduce reporting bias, data collection was completed before any assessments of quality were performed. In studies reporting data for more than one experimental group, both sets of data were included if different materials were used with the same peptide. In studies where multiple concentrations of the peptide were used, the highest concentration was selected. In the conduct of this systematic review, no discrepancies between reviewers in study inclusion nor extracted data points were identified, with 100% concordance between the reviewers and hence inter‐rater reliability was not formally assessed.

### 2.4 Risk of Bias Assessment

The risk of bias (RoB) was assessed independently by two reviewers (LW and ZA), using the SYRCLE RoB tool, specifically developed for animal studies [16]. The tool assesses ten domains, including (1) sequence generation, (2) baseline characteristics, (3) allocation concealment, (4) random housing, (5) blinding (performance bias), (6) random outcome assessment, (7) blinding (detection bias), (8) incomplete outcome data, (9) selective outcome reporting, and (10) other sources of bias. Following assessment by two independent reviewers, results were compared. In the case of discrepancies, we planned for further authors to provide critical appraisal of the studies followed by open discussion and voting. However, no discrepancies in the risk of bias were identified, and hence such steps to achieve consensus were not required.

### 2.5 Data Synthesis and Statistical Analysis

Where raw data was unavailable, data points and standard deviations were extracted from figures of the included studies using WebPlotDigitizer Version 4 (https://automeris.io/). In studies that reported percentage wound size rather than healing rates, data was converted by subtracting wound size percentage from 100. Extracted data was used to perform meta‐analyses on the size of wounds at the relevant timepoints. RevMan 5.4 (Cochrane Informatics & Technology, London, UK) software was used for all meta‐analyses; meta‐analyses were completed according to timepoints and were carried out on all studies that reported wound size at the respective timepoint (at 3, 7, 10 and 14 days after injury). A minimum of three studies were required to perform a meta‐analysis. Following an initial analysis and after identifying its heterogeneity, further meta‐analyses were completed by dividing studies according to the species used in the animal model. A random effects model was applied. *Q* and *I* ^2^ statistics were used to determine heterogeneity in the studies, with an *I* ^2^ greater than 50% being categorised as considerable heterogeneity. In all cases, the carrier material without AMPs was used as the control group in all meta‐analyses and results were reported as mean difference (MD).

## 3 Results

### 3.1 Study Selection

**FIGURE 1:**
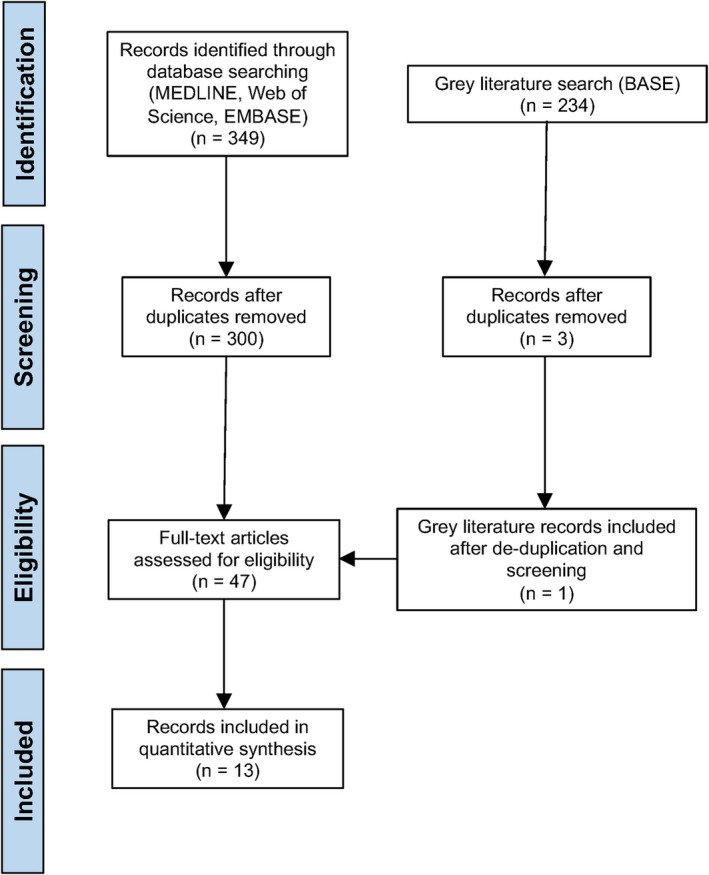
PRISMA Flow Chart of Study Selection.

The search results are outlined in the PRISMA flow diagram (Figure 1). A total of 583 studies were identified through database and grey literature searches. After duplicates were removed, 303 studies remained, of which 48 were included in the full‐text screening. Following this, thirteen studies remained and were included in this systematic review. Whilst meeting the inclusion and exclusion criteria, one study was excluded during the data extraction phase of the review since the study used just 3 animals in the wound healing model but inflicted three wounds per animal [17]. Whilst conforming to the 3Rs principle of ethical animal research, namely replacement, reduction, and refinement, by reducing the number of animals involved in the study, the work was judged as being inappropriate for inclusion in the review due to the wound burden per animal being substantially higher than in all other included studies. In addition, another study conformed to all inclusion criteria, but numerical data for wound healing was not reported in sufficient detail to allow inclusion in the quantitative analyses [18]. This was not resolved upon requesting data from authors, and hence the study was excluded from the meta‐analysis due to the lack of quantitative data but was included in the qualitative synthesis. Furthermore, another study was excluded due to the antimicrobial agent and drug delivery systems being in a liquid form, as all other included studies that used agents incorporated into either solid or hydrogel systems. A liquid‐based therapy was thought to have substantially different therapeutic release kinetics, and thus would introduce significant heterogeneity into the experimental design [19].

Of the included studies, two investigated different materials with the addition of AMPs: Huang et al. compared hydroxypropylcellulose and sodium alginate formulations with and without AMP [20], and Wang et al. compared carbomer and chitosan formulations with and without AMP [21]. Similarly, Ni et al. included a study group with and without the addition of angiogenic peptide as well as an AMP; hence both study groups were included [22]. Three studies reported the use of different concentrations of peptides in their formulations [20, 23, 24], however only the highest concentration of peptide was included in the review to avoid bias from manually selecting a concentration to analyse and to avoid including multiple groups of the same study in one meta‐analysis, which may skew the data. Three studies included a more complex combination of formulations [25, 26, 27], with Lei et al. [26] including study groups with photodynamic peptides exposed to photo‐irradiation. However, as photodynamic therapeutics warrant a separate review, the effect of the photo‐irradiation was not included in this systematic review.

### 3.2 Study Characteristics

Of the included studies, twelve were performed in China [20, 21, 22, 23, 24, 25, 26, 27, 28, 29, 30, 31] and one was performed in South Africa [32]. Seven studies were performed in BALB/c mice [20, 21, 24, 26, 27, 28, 32] whilst one was performed in C57BL/6 mice [23]. Five studies were performed in Sprague–Dawley rats [22, 25, 29, 30, 31] and a mixture of male and female animals was used. The materials used in the studies varied from nanofibers and hydrogels to microneedle patches and films, and a breadth of AMPs were incorporated into these materials (Table 2 and Table S1). The study groups for the infected wound model included as a minimum, the material with AMPs, and the blank material without AMPs in all cases. In one of the studies, different descriptions for the control groups were used throughout the study, causing some ambiguity in what treatment was administered to each group [20].

**TABLE 2:** Characteristics of included studies.

Study	Origin	Antimicrobial peptide name	Carrier material	Species/Strain	Sex	Age (weeks)	Study groups	*n*/Group
Fan et al. 2024 [23]	China	KGRT (Lys‐Gly‐Arg‐Thr)	self‐healing hydrogel containing oxidised dextran, adipic acid dihydrazide and alkylated oxidised dextran	Mouse, C57BL/6	Male	8	GEL‐C (oxidised dextran (OD) + adipic acid dihydrazide (ADH) + alkylated oxidised dextran (DOD) 150 μg/mL KGRT); GEL‐D (OD + ADH + DOD + 200 μg/mL KGRT); control; GEL‐5 (OD + ADH + 0.10 g/mL DOD)	6
Heunis et al. 2013 [32]	South Africa	nisin	Polyethylene oxide and poly (D,L‐lactide) nanofibers	Mouse, BALB/c	Male	adult	Nisin‐containing nanofibers nisin‐free nanofibers	7
Huang et al. 2022 [20]	China	NZ2114 (a plectasin‐derived peptide)	Sodium carboxymethylcellulose, hydroxypropyl cellulose, and sodium alginate hydrogel	Mouse, BALB/c	Female	6–8	HPC/SA‐NZ2114 hydrogel 0.512 mg/g; HPC/SA‐NZ2114 hydrogel 1.024 mg/g; No infection or treatment—blank control; Infected with blank HPC/SA hydrogel—negative control; Mupirocin—positive control; Ofloxacin—positive control	6
Hussain et al. 2021 [28]	China	Dopamine‐substituted multidomain peptide (DAP)	Self‐assembled nanofibers	Mouse, BALB/c	Female	/	DAP hydrogel; untreated control hydrocolloid	8
Lei et al. 2020 [26]	China	AMP_2_	Gelatin‐based composite hydrogel	Mouse, BALB/c	Female	6	Gel–Col@AMP_2_‐Ce6 hydrogel without photo‐irradiation; Gel–Col@AMP_2_‐Ce6 hydrogel with photo‐irradiation; PBS; Gelatin; AMP_2_‐Ce6 without photo‐irradiation; AMP_2_‐Ce6 with photo‐irradiation; Gel–Col hydrogel only	5
Wang et al. 2023 [27]	China	KKLRLKIAFK	Chitosan and gum arabic nanogel microneedle patches	Mouse, BALB/c	/	/	PVP + Col III; PVP + Col III + CG‐NPs; PVP + Col III + AMP‐Cy3; PVP + Col III + CGA‐NPs; MN/PVP + Col III + CGA‐NPs	3
Wang Guixi et al. 2024 [21]	China	Cathelicidin‐DM	Carbomer and chitosan	Mouse, BALB/C	Male	/	DM‐carbomer hydrogel; DM‐chitosan hydrogel; control; ampicillin; cathelicidin‐DM; carbomer hydrogel; chitosan hydrogel	5
Wang Y et al. 2024 [24]	China	Cathelicidin‐BF	Hyaluronan hydrogel scaffold	Mouse, BALB/C	Male	/	HAG‐g‐C2; HAG‐g‐C5; blank; HAG	3
Li et al. 2023 [29]	China/UK	C_8_‐G (IIKK)_2_I‐NH_2_ (C_8_G_2_)	β‐glucan aldehyde hydrogel	Rat, SD	Male	/	C_8_G_2_; BG; BGA/C_8_G_2_	3
Lin et al. 2023 [30]	China	Plantaricin 149	Hyaluronic acid hydrogel	Rat, SD	Female	/	HAD@AMP; HAD; control	8
Ni et al. 2024 [22]	China	melittin	Au‐S‐based hydrogel	Rat, SD	Male	6–8	Au‐S/MS‐KS hydrogel; Au‐S/MS hydrogel; control; Au‐S hydrogel; Au‐S/KS hydrogel	5
Wang Guanyi et al. 2024 [25]	China/USA	CRRI3	GelMA microneedles	Rat, SD	/	/	GMCM+NIR; control; 3 M dressing; GMCM	6
Zhou et al. 2023 [31]	China	Actinomycin‐X2	Silk fibroin film	Rat, SD	Male	4–6	Silk fibroin film containing 5 ug/mL Ac.X2; gauze only; dressing with no Ac.X2	6

*Note:* Brackets indicate sequence determined via literature review.

Abbreviations: /, Not reported; AMP, Antimicrobial peptide; AMP_2_, Antibacterial photodynamic peptide; AMP‐Cy3, Cyanine 3 labelled AMP; Au‐S, Gold‐thiol (S‐S bonds); Au‐S/MS‐KS hydrogel, Gold‐thiol/Melittin‐keratinocyte‐stimulating peptide‐based hydrogel; Au‐S/KS hydrogel, Gold‐thiol/Keratinocyte‐stimulating peptide‐based hydrogel; Au‐S/MS hydrogel, Gold‐thiol/Melittin‐based hydrogel; BG, β‐D‐glucan; BGA, Oxidised β‐D‐glucan; Ce6, Chlorin e6 (photosensitiser); CG, Galactose‐based glycopolymer; CGA, Chlorogenic acid; Coll III, Collagen III; DAP, Dopamine‐substituted multidomain peptide; FT, Full thickness; Gel–Col@AMP2‐Ce6, Gelatin‐based composite hydrogel; GelMA, Methacrylated gelatin; GMCM, Methacrylated gelatin (GelMA) + AMP + hollow MnO_2_ nanoparticles; GMCM+NIR, GMCM+Near infrared light; HAD, Hyaluronic acid‐based asymmetric‐adhesive (Janus) hydrogel; HAG, Hyaluronan scaffold gel; HAG‐g‐C2, Hyaluronan scaffold gel, crosslinked with gallic acid modified gelatin and cathelicidin 2; HAG‐g‐C5, Hyaluronan scaffold gel, crosslinked with gallic acid modified gelatin and cathelicidin 5; HPC, Hydroxypropyl cellulose; MN, Microneedle; NP, Nanoparticles; PVP, Poly (vinylpyrrolidone); SA, Sodium alginate; SD, Sprague–Dawley.

### 3.3 Animal Model Wound Characteristics

In terms of wound creation, most frequently, a circular, full‐thickness wound was created with a diameter of 10 mm on the dorsum of the animal [20, 23, 29, 30, 31]. However, some studies used a smaller diameter wound [21, 28, 32], a larger diameter wound [22, 25], or wounds with an oval or square shape (Table S3) [25, 26, 27].

Where reported, the research groups administered bacteria at a concentration of 1 × 10^6^ to 1 × 10^8^ CFU/mL [20, 21, 22, 23, 24, 26, 27, 28, 29, 30, 31, 32] and a volume of 53.3 ± 62.0 μL to infect the wounds [20, 21, 22, 23, 28, 29, 30, 31, 32]. Of the included studies, one applied bioluminescent *S. aureus* to enable bioluminescent imaging of the inflicted wounds as a means of quantifying viable bacteria [32]. Following wound creation and infection, wounds were exposed to the treatments for 12.85 ± 3.39 days, with the most commonly used endpoint being 12 days (Table S3).

### 3.4 Risk of Bias Assessment

**FIGURE 2:**
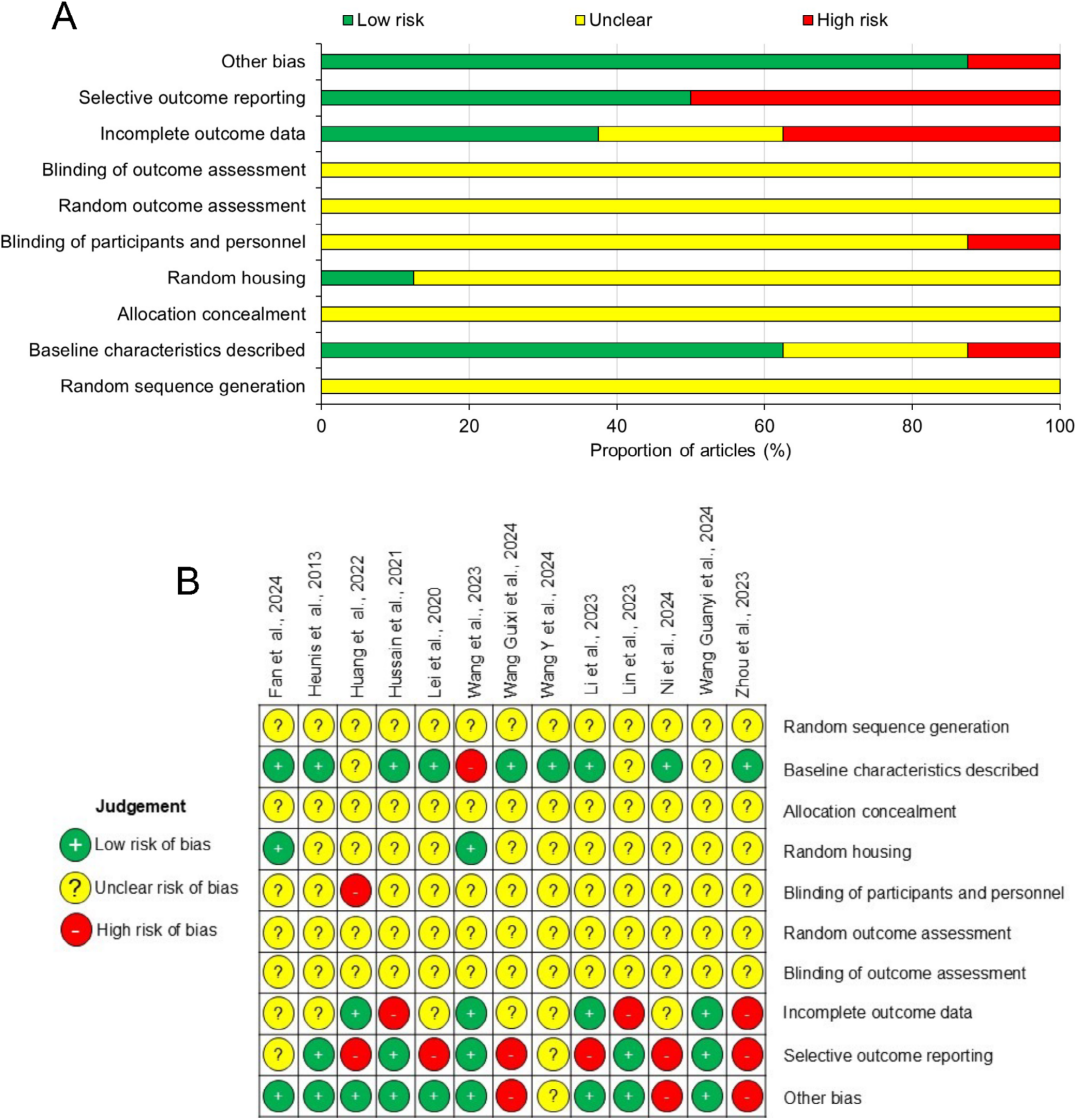
Risk of Bias in the Included Studies. (A) Summary chart to show risk of bias in the included studies. (B) Risk of bias in individual studies.

The risk of bias analysis using the SYRCLE risk of bias tool showed that all domains contained some unclear or high risk of bias (Figure 2A,B). For example, in domains for blinding of outcome assessment, random outcome assessment, allocation concealment, and random sequence generation, 100% of studies contained unclear risk of bias. Selective outcome reporting contained 50% of studies with high risk of bias, while 38% and 13% of studies in domains assessing incomplete outcome data, other bias, blinding of participants and personnel, and baseline characteristics described contained high risk of bias, respectively.

### 3.5 Synthesis of Results From Included Studies

#### 3.5.1 Wound Healing Rate

In all of the included studies, except one, materials incorporated with AMPs were either reported as superior or promoted significantly improved wound closure over the different experimental time points compared to AMP‐free materials [20, 21, 22, 23, 24, 25, 26, 27, 28, 29, 30, 31]. The one exception was a study which used nanofibers, with and without AMPs, which was reported to stimulate wound closure and significantly reduced *S. aureus* Xen 36 cell viability and bioluminescence within the wound bed, indicating capacity to reduce bacterial burden in wounds, but no significant difference in wound healing could be identified between nanofibers with and without peptides incorporated into them [32].

**FIGURE 3:**
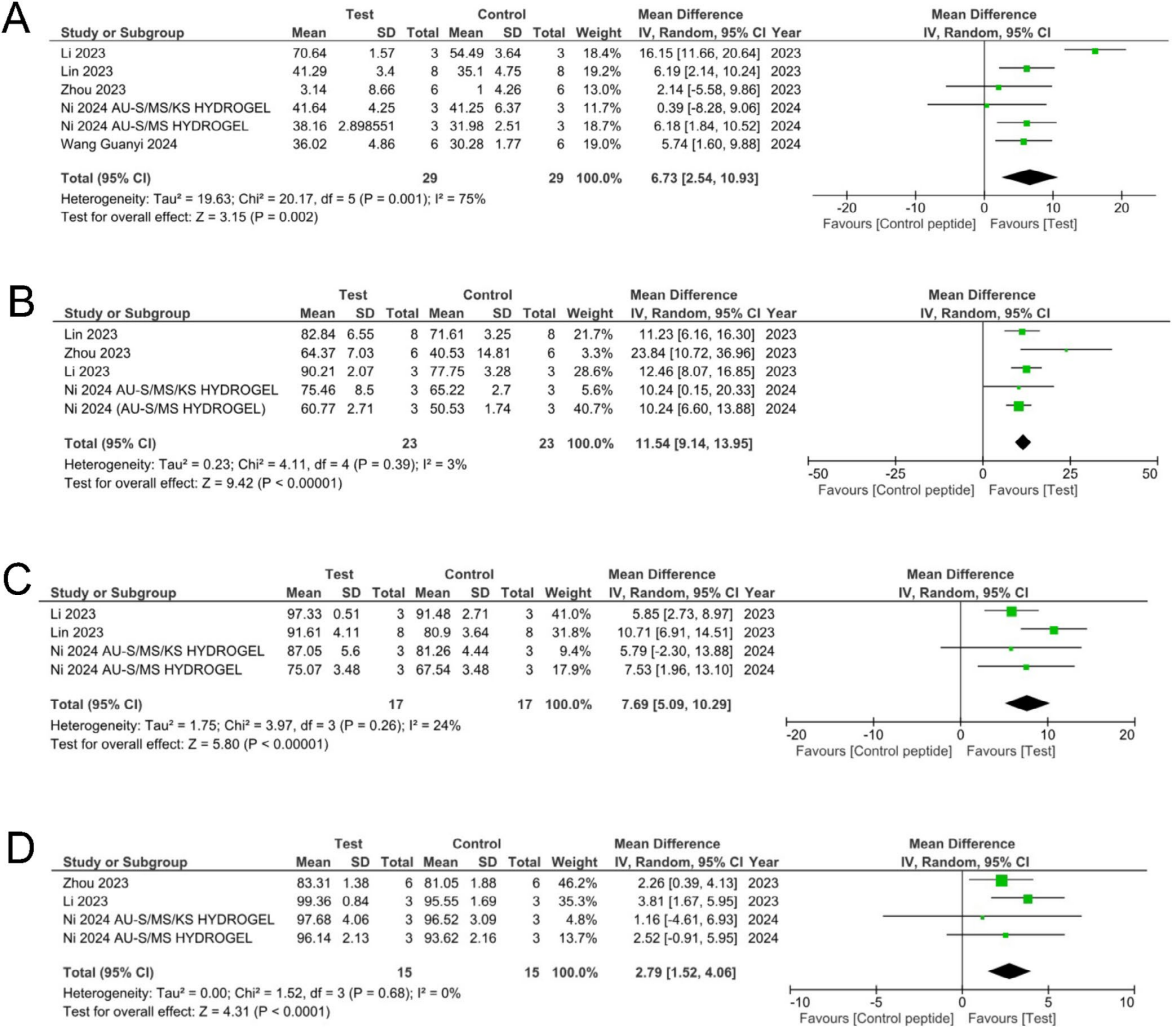
Meta‐analysis of pooled data for wound healing rates in rats over time. Forest plots to show wound healing rates at (A) 3, (B) 7, (C) 10, and (D) 14 days after injury and treatment.

Meta‐analyses of pooled data for wound healing rates in rats showed significantly improved wound healing at 3 days (MD = 6.73%, 95% CI [2.54%, 10.93%], *p* = 0.002), 7 days (MD = 11.54%, 95% CI [9.14%, 13.95%], *p* < 0.00001), 10 days (MD = 7.69%, 95% CI [5.09%, 10.29%], *p* < 0.00001) and 14 days (MD = 2.79%, 95% CI [1.52%, 4.06%], *p* < 0.0001) post‐treatment in favour of the test AMP over the control dressing (Figure 3).

In mouse studies, pooled data used to perform meta‐analyses showed that AMP‐loaded dressing materials significantly improved wound healing rates at the 7 day, 10 day, and 14 day timepoints, with the most significant improvement observed at the final timepoint after 14 days of treatment (MD = 32.30%, 95% CI [18.06%, 46.54%], *p* < 0.00001; Figure S1).

When the data for both mouse and rat were combined, wound healing rates at 3, 7, 10 and 14 days after injury remained significantly improved in favour of the treatment (Figure S2).

### 3.6 Qualitative Analysis

#### 3.6.1 Histological Assessment

A variety of histological assessments were made by the thirteen included studies. This included quantification of epidermal thickness, % wound re‐epithelialisation, granulation tissue thickness, collagen deposition, new blood vessels, CD31 staining and localisation of growth factors such as vascular endothelial growth factor (VEGF), epidermal growth factor (EGF) and pro‐ and anti‐inflammatory cytokines (e.g., tumour necrosis factor (TNF)‐α and interleukin (IL)‐6 and IL‐10). AMP‐incorporated materials in general reduced the localisation of proinflammatory cytokines (Table 3) [22, 24, 30] and upregulated anti‐inflammatory cytokines (Table 3) [22, 24]. The addition of AMPs to dressings also increased the localisation of the growth factors VEGF and transforming growth factor (TGF)‐ß (Table 4) [20, 22, 24, 25, 30]. Though, of the histological analyses, the strongest evidence base has crystallised for AMPs enhancing angiogenesis, with five studies reporting an increase in the marker for angiogenesis, CD31 [22, 23, 25, 26, 27], and a further two studies reporting an increase in blood vessel formation (Table 5) [28, 30]. A large number of the studies also reported an increase in collagen deposition as a result of the addition of AMPs [21, 22, 25, 26, 27, 28, 30], as well as some showing an increase in epidermal thickness [22, 30], hair follicle formation [29] and granulation tissue formation (Table 6) [22].

**TABLE 3:** Summary of histological data for pro‐ and anti‐inflammatory cytokines in included studies.

Study	Marker					
	IL‐1β	TNF‐α	CD86	IL‐10	IL‐6	CD206
Fan et al. 2024 [23]; Gel‐D (D 14)	NS*	−−−*				
Wang Y et al. 2024 [24]; HAG‐g‐C5 (D 12)		−−−	−−−			+++
Lin et al. 2023 [30]; (D10)		−*				
Ni et al. 2024 [22]; Au‐S/MS hydrogel (D 14)		−−*		+*		
Ni et al. 2024 [22]; Au‐S/MS/KS hydrogel (D 14)		NS*		+*		
Hussain et al. 2021 [28]					NS	
Li et al. 2023 [29]					−*	

*Note:* Data is only included for studies which reported changes in an appropriate marker. Changes in levels of growth factors in test group compared to peptide‐free controls at the final timepoint of study (in brackets). − slightly reduced expression *p* ≤ 0.05; −− moderately reduced expression *p* ≤ 0.01; −−− strongly reduced expression *p* ≤ 0.001; −−−− very strongly reduced expression *p* ≤ 0.0001; + slightly increased expression *p* ≤ 0.05; ++ moderately increased expression *p* ≤ 0.01; +++ strongly increased expression *p* ≤ 0.001; ++++ very strongly increased expression *p* ≤ 0.0001; ns, no significant difference; * estimated because no significance details provided, D, day.

**TABLE 4:** Summary of histological data for growth factor expression in included studies.

Study	Marker		
	TGF‐β	VEGF	EGF
Huang et al. 2022 [20]; HPC‐NZ2114		++++	NS
Huang et al. 2022 [20]; SA‐MZ2114		+	NS
Wang Y et al. 2024 [24]; HAG‐g‐C2	+++		
Wang Y et al. 2024 [24]; HAG‐g‐C5	+++		
Lin et al. 2023 [30]	+		
Ni et al. 2024 [22]; Au‐S/MS hydrogel		+*	
Ni et al. 2024 22; Au‐S/MS/KS hydrogel		+*	
Wang Guanyi et al. 2024 [25]	+++		

*Note:* Data is only included for studies which reported changes in an appropriate marker. Changes in levels of pro‐inflammatory and anti‐inflammatory cytokines and molecules in test group compared to peptide‐free controls at the final timepoint of study (in brackets). − slightly reduced expression *p* ≤ 0.05; −− moderately reduced expression *p* ≤ 0.01; −−− strongly reduced expression *p* ≤ 0.001; −−−− very strongly reduced expression *p* ≤ 0.0001; + slightly increased expression *p* ≤ 0.05; ++ moderately increased expression *p* ≤ 0.01; +++ strongly increased expression *p* ≤ 0.001; ++++ very strongly increased expression *p* ≤ 0.0001; ns no significant difference; * estimated because no significance details provided, D = day.

**TABLE 5:** Summary of histological data for other histological markers in included studies.

Study	Marker			
	Ki67	CD31	Col‐I	Coll‐II
Fan et al. 2024 [23]; Gel‐D		++++		
Huang et al. 2022 [20]; HPC‐NZ2114		−−−−		
Huang et al. 2022 [20]; SA‐MZ2114		NS		
Hussain et al. 2021 [28]				
Lei et al. 2020 [26]		+++		
Wang et al. 2023 [27]		+++*		
Wang Guixi et al. 2024 [21]; DM‐carbomer				
Wang Guixi et al. 2024 [21]; DM‐chitosan				
Li et al. 2023 [29]				
Lin et al. 2023 [30]		+*		
Ni et al. 2024 [22]; Au‐S/MS hydrogel		++*		
Ni et al. 2024 [22]; Au‐S/MS/KS hydrogel		++*		
Wang Guanyi et al. 2024 [25]	+++	+++	+++	+++

*Note:* Data is only included for studies which reported changes in an appropriate marker. Changes in levels of other markers in test group compared to peptide‐free controls at the final timepoint of study (in brackets). − slightly reduced expression *p* ≤ 0.05; −− moderately reduced expression *p* ≤ 0.01; −−− strongly reduced expression *p* ≤ 0.001; −−−− very strongly reduced expression *p* ≤ 0.0001; + slightly increased expression *p* ≤ 0.05; ++ moderately increased expression *p* ≤ 0.01; +++ strongly increased expression *p* ≤ 0.001; ++++ very strongly increased expression *p* ≤ 0.0001; ns, no significant difference; * estimated because no significance details provided, D, day.

**TABLE 6:** Summary of histological features of wound healing in included studies.

Study	Histological markers						
	Collagen deposition	Epidermis thickness	Granulation tissue	Wound re‐epithelialisation	Hair follicle formation	Blood vessel formation	Relative newborn skin
Hussain et al. 2021 [28]	+			+		++	
Lei et al. 2020 [26]	++						++++
Wang et al. 2023 [27]	+++*						+++*
Wang Guixi et al. 2024 [21]; DM‐carbomer	+*						
Wang Guixi et al. 2024 [21]; DM‐chitosan	+*						
Li et al. 2023 [29]					+*		
Lin et al. 2023 [30]	+	+				+	
Ni et al. 2024 [22]; Au‐S/MS hydrogel	+++	+++	+++				
Ni et al. 2024 [22]; Au‐S/MS/KS hydrogel	NS	NS	NS				

*Note:* Data is only included for studies which reported changes in an appropriate marker. Changes in levels of wound healing‐related factors in test group compared to peptide‐free controls at the final timepoint of study (in brackets). − slightly reduced expression *p* ≤ 0.05; −− moderately reduced expression *p* ≤ 0.01; −−− strongly reduced expression *p* ≤ 0.001; −−−− very strongly reduced expression *p* ≤ 0.0001; + slightly increased expression *p* ≤ 0.05; ++ moderately increased expression *p* ≤ 0.01; +++ strongly increased expression *p* ≤ 0.001; ++++ very strongly increased expression *p* ≤ 0.0001; ns, no significant difference; * estimated because no significance details provided, D, day.

#### 3.6.2 Antimicrobial Properties of AMP‐Containing Materials

Microbial growth was reported at a range of timepoints from 0.5 to 21 days after the addition of the dressings [20, 23, 26, 28, 29, 31, 32], with two of these studies reporting microbial growth at unknown timepoints [21, 25]. One of the included studies reported microbial growth on both the dressing and the wound, providing a comprehensive analysis of bacterial presence during treatment and demonstrating a strongly significant reduction in both the dressing and wound [32]. As expected, of the studies that reported microbial growth during the wound healing experiment, all reported that the addition of AMPs to dressings strongly reduced the presence of bacteria in the wounds.

Across the included studies, a wide range of distinct AMPs were evaluated, with no AMP investigated across multiple independent manuscripts. Consequently, the heterogeneity in AMP type, experimental design, and reported outcomes precluded meaningful comparative analysis or identification of consistent relationships between AMP characteristics and study outcomes.

## 4 Discussion

### 4.1 Summary of Findings

The objective of this review was to identify whether dressing materials containing AMPs had a beneficial effect on the wound healing of *S. aureus* ‐infected wounds compared to dressings lacking AMPs. This review, however, did not aim to rank individual antimicrobial peptides across formulations, as efficacy is strongly influenced by delivery modality, dosing, and exposure time, which vary substantially between topical solutions, dressings, and other application strategies. Overall, the results of the meta‐analyses strongly suggest that AMP‐containing dressings significantly enhance the wound healing rate compared to AMP‐free dressings, as demonstrated at the 3‐day and 14‐day timepoints in rat studies and the 7‐day, 10‐day and 14‐day timepoints in mouse studies. An increasingly strong effect is seen as time progresses, indicating that AMP‐containing dressings reduce time to healing compared to AMP‐free materials. Meta‐analyses completed on the combined pool of rat and mouse studies further mirrored this observation (Figure S1). However these analyses must be interpreted with caution due to the high heterogeneity introduced by the two distinct model species.

Histologically, these effects appear to be mediated by down‐regulation of pro‐inflammatory cytokines, upregulation of anti‐inflammatory cytokines such as IL‐10 and CD206, and increased expression of growth factors including TGF‐β and VEGF. In tandem, the histological data of included studies indicate that the presence of AMPs has a positive effect on collagen deposition and angiogenesis, together culminating in more efficient wound healing of the *S. aureus* ‐infected wounds. An astonishingly strong impact from AMPs was seen in angiogenesis, with seven studies corroborating that the presence of AMPs promoted new blood vessel formation [22, 23, 25, 26, 27, 28, 30].

### 4.2 Explanation of the Findings

Aside from the increased wound healing potential seen when incorporating AMPs into the dressing materials, a clear reduction in pathogenic load was also confirmed in all studies reporting microbial counts. This is especially interesting in the context of studies investigating MRSA‐infected wounds, further establishing AMP‐containing dressings as an alternative treatment pathway to conventional antibiotics in the treatment of infected wounds [20, 29].

The inclusion and exclusion criteria were designed to ensure a robust dataset capable of supporting meaningful conclusions. Rodent models were included because they are among the most commonly used animal modelsand provide a benchmark for further studies. Therefore, a comparatively large number of relevant studies could be selected for the review, a factor which was identified in preliminary searches. *S. aureus‐*infected wounds were chosen due to their vast abundance in the clinical setting and thus their substantial clinical relevance [33, 34]. The application of dressing materials containing AMPs was chosen due to the increasing interest shown toward AMP‐based formulations currently [35, 36, 37]; liquid solutions were excluded from the review due to the likely variation in mechanisms of action and their application to the wound. For example, whilst ointments often require frequent re‐application, the dressings are applied for longer timepoints due to their increased stability. Similarly, ointments are often designed to provide moisture and enable direct delivery of the therapeutic agent at the core of their development [38], whilst dressing materials also serve the purpose of protection of the wound bed and providing a physical barrier [39]. Inclusion of these approaches would have introduced substantial additional heterogeneity to an already diverse evidence base.

Despite being excluded from the meta‐analysis on the premise of using a liquid‐based drug delivery system, research by Mei et al. [19] determined that the wound healing properties of graphene oxide and polyethylene glycol nanoparticles could induce significantly greater wound healing properties compared to a negative control when the antimicrobial peptide OH30 was added to the formulation, further supporting the evidence provided by the studies included in the analysis. Here, it was identified that both peptide‐free and peptide‐loaded nanoparticles engendered close to 100% wound recovery within the 14 days of the experiment, but that the addition of the AMP expedited the rate at which this was attained. In addition, the AMP component of the nanoparticles appeared to reduce the presence of *S. aureus* in the wound bed by the 14‐day timepoint, although no comment was made regarding its statistical difference compared to the peptide‐free nanoparticles. This reflects a similar trend to that seen in the solid dressing counterparts described in the meta‐analysis.

Moreover, a study by Yu et al. [18] was excluded from the meta‐analysis because numerical data was not accessible. However, this group also concluded that the addition of AMPs accelerated wound healing by visually inspecting the size and appearance of the wound, with the AMP‐containing dressing healing without a scar by the 14th day whilst the peptide‐free dressing treated wounds had not yet fully healed, supporting the hypothesis that AMPs within dressings promote wound healing to a higher degree than AMP‐free dressing materials [18].

This systematic review did not aim to rank individual AMPs across formulations, as efficacy is strongly influenced by delivery modality, dosing, and exposure time, which vary substantially between topical solutions, dressings, and other application strategies. However, future studies should integrate systematic evaluation of AMP identity with delivery‐format optimisation to determine how peptide selection and formulation collectively influence antimicrobial efficacy and wound healing outcomes.

### 4.3 Translational Relevance to Human Wound Healing

Although this review focused exclusively on controlled rodent models of *S. aureus* –infected excisional wounds, the findings are relevant to the ongoing development of antimicrobial dressings for human use. In clinical practice, antimicrobial dressings are widely employed as adjunctive therapies to control bioburden and create conditions favourable for healing, yet robust clinical evidence for AMP‐based dressings remains limited and heterogeneous [40, 41]. This likely reflects, in part, the complexity of human wounds, which vary substantially in aetiology, chronicity, host comorbidities, and microbial burden.

Importantly, several translational challenges identified in human studies, including peptide stability, dosing strategies, wound exposure time, and safety considerations, are directly informed by the mechanistic outcomes assessed in preclinical models, such as modulation of inflammation, angiogenesis, and bacterial clearance [40, 41]. However, differences in healing mechanisms between rodents and humans, particularly the predominance of wound contraction in rodents compared with re‐epithelialisation in humans, limits direct extrapolation of efficacy outcomes [42, 43].

The heterogeneity and methodological limitations identified in the preclinical literature by this review may therefore contribute to the variable success of AMP‐based dressing strategies in clinical translation. Addressing these gaps through improved standardisation, adherence to reporting guidelines, and clinically relevant outcome measures in animal studies will be critical to supporting the progression of AMP‐loaded dressings toward meaningful human application [40, 44].

### 4.4 Limitations

A number of limitations have hampered the quality of evidence provided, despite the significant findings. Firstly, two different rodent species were used. Rats and mice differ anatomically in epidermal thickness, as well as in size and body mass. Because of this, the relative size of the inflicted wounds is different in the two animal species. In addition, there are undeniable differences in the wound healing between these species as well as compared to humans; the primary mode of wound closure is by contraction in both rats and mice, compared to re‐epithelialisation in humans. However, these differences were mitigated through the inclusion of analyses that segregated data from rats and mice.

Whilst using separate analyses of rats and mice reduced the heterogeneity of meta‐analyses, in some cases a high heterogeneity was identified nonetheless. This is due to indisputable differences in methodologies between studies; the included studies applied a broad spectrum of materials and antimicrobial peptides. The application of different polymeric materials likely results in variation in degradation behaviour and mechanisms of disintegration. Consequently, drug release profiles and modes of action also differ between studies. Additionally, there were variations in how the wounds were inflicted and the bacterial burden, resulting in variability in wound characteristics across studies. Similarly, the strain of *S. aureus* used for inducing infection varied, with some studies using multidrug‐resistant strains and others using susceptible strains, contributing to a greater heterogeneity in experimental outcomes. Contrastingly, it may be argued that by including studies using different strains, the data becomes more reflective of the breadth of pathogens faced in the clinical setting.

Aside from this, there were also variations in timepoints used for monitoring the outcomes, parameters used for monitoring them, and how these were reported; for example, whilst the majority of studies reported histological observations at the final timepoint, one study reported immunohistochemical and immunofluorescence staining on day 3, granulation tissue formation and collagen deposition on day 7, and hair follicle formation on day 14, with no justification for the selected timepoints. For this reason, it could not be included in the histological analysis of the review [29]. The timepoints used also varied between studies more generally, reducing the number of studies included in each of the meta‐analyses and therefore also the power of the data. In future, more standardised methods for timepoint selection and wound creation should be established to permit greater comparability between studies.

A key limitation of the current evidence base is the marked heterogeneity in the AMPs studied, with most investigations focusing on distinct peptides evaluated in isolation and without replication across independent studies. This lack of convergence limits the ability to draw robust conclusions regarding comparative efficacy or to identify consistent associations between AMP structural class, mode of action, and observed outcomes. In addition, variability in experimental models, outcome measures, and reporting further constrains synthesis and reduces the feasibility of stratified or mechanistic comparisons. As such, any inference regarding structure–function–outcome relationships would be speculative, and we have therefore avoided overinterpretation of these data.

A further limitation relating to the animal models used is the lack of adherence to ARRIVE guidelines which may lead to an increased risk of bias from researchers; by failing to appropriately randomise groups, mitigate for differences in cage conditions and blind assessors to the treatments of the animals, there is an increased risk of introducing detection and assessment biases. The included studies involved a sample size of between 3 and 8 individuals, which are generally low sample sizes. In addition to this, a small number of studies fit the inclusion criteria of the study and hence the overall power of the systematic review and meta‐analyses was limited. Especially the *I* ^2^ statistic used for evaluating heterogeneity can be biased in smaller meta‐analyses; therefore, the presented data must be interpreted with caution [40].

Whilst our search strategy could have been broadened and the exclusion criteria relaxed to include additional studies, our approach was intentionally targeted to address a specific question: the efficacy of AMP‐loaded dressings in *S. aureus* ‐infected rodent wound models. Although this resulted in a relatively small number of included studies, it reflects the limited and heterogeneous nature of the current evidence base rather than overly restrictive criteria. Expanding the inclusion criteria would substantially increase heterogeneity in intervention, delivery mechanism, and outcome measures, thereby limiting the ability to draw meaningful or clinically relevant conclusions specific to AMP‐based dressings.

## 5 Conclusion

Despite the low number of included studies and small sample sizes, the meta‐analyses indicate a strong positive effect of incorporating AMPs into dressing materials. We suggest that adding AMPs to dressing materials increases the rate of wound healing in *S. aureus* ‐infected wounds, given that it is among the most prevalent pathogens in wound infections. Our systematic review confirms that AMPs bestow strong antimicrobial properties upon the dressing materials and offer evidence that they enhance the wound healing process by upregulating anti‐inflammatory cytokines and growth factors, downregulating pro‐inflammatory cytokines, and encouraging angiogenesis within the wound bed. We highlight the importance of adherence to the ARRIVE guidelines and greater standardisation across methods, and we observe the growing interest in using AMPs for antimicrobial dressing materials.

## Author Contributions

Conceptualization: L.R., A.S., S.A.K., M.M.G., Z.A. Study design: L.R., A.S., S.A.K., M.M.G., Z.A., Generated the data: L.R., Z.A. Data analyses: L.R., Z.A. Writing – original draft: L.R., Z.A. Writing – review and editing: L.R., A.S., S.A.K., M.M.G., Z.A.

## Funding

This work was supported by the University of Birmingham's College of Engineering and Physical Sciences (PATH mini CDT).

## Conflicts of Interest

Z.A. is a co‐founder and co‐owner of Midland Pharmaceuticals, which is a clinical stage company developing treatments for spinal cord injury, unrelated to this study. All other authors declare no conflicts of interest.

## Supporting information


**Data S1:** wrr70183‐sup‐0001‐DataS1.docx.

**Table S1:** Full search terms for all databases.
**Table S2:** Details of Antimicrobial Peptide Sequences Used in the Included Studies. Brackets indicate sequence determined via literature review.
**Table S3:** Wound Characteristics. CFU, colony forming units; FT, full thickness; MRSA, methicillin resistant *S. aureus.*

**Figure S1:** Meta‐analysis of pooled data for wound healing rates in mice over time. Forest plots to show wound healing rates at (A) 7, (B) 10, and (C) 14 days after injury and treatment.
**Figure S2:** Meta‐analysis of pooled data for wound healing rates in rats and mice combined over time. Meta‐ANALYSIS shows wound healing rates at (A) 3, (B) 7, (C) 10 and (D) 14 days after injury and treatment.

## Data Availability

The data that support the findings of this study are available from the corresponding author upon reasonable request.
