# Supplementary material for: In Vivo Wound Healing Effects of Antimicrobial Peptide‐Based Dressings for S. aureus‐Infected Wounds—A Systematic Review and Meta‐Analysis
Source: Wound Repair Regen. 2026 Jun 22;34(3):e70183. doi: 10.1111/wrr.70183 (PMC13287310; doi:10.1111/wrr.70183)
Supplement: Supplementary file 2 — Table S1: Full search terms for all databases. Table S2: Details of Antimicrobial Peptide Sequences Used in the Included Studies. Brackets indicate sequence determined via literature review. Table S3: Wound Characteristics. CFU, colony forming units; FT, full thickness; MRSA, methicillin resistant S. aureus. Figure S1: Meta‐analysis of pooled data for wound healing rates in mice over time. Forest plots to show wound healing rates at (A) 7, (B) 10, and (C) 14 days after injury and treatment. Figure S2: Meta‐analysis of pooled data for wound healing rates in rats and mice combined over time. Meta‐ANALYSIS shows wound healing rates at (A) 3, (B) 7, (C) 10 and (D) 14 days after injury and treatment. [file WRR-34-0-s001.docx]

**SUPPLEMENTARY INFORMATION**

***IN VIVO* WOUND HEALING EFFECTS OF ANTIMICROBIAL PEPTIDE-BASED DRESSINGS FOR *S. AUREUS-*INFECTED WOUNDS – A SYSTEMATIC REVIEW AND META-ANALYSIS**

Lea Wood, MSc^1^, Artemis Stamboulis, PhD^1^, Sarah A. Kuehne, PhD^2^, Melissa M. Grant, PhD^3,4,5^*, Zubair Ahmed, PhD^,6,7,8^*

^1^School of Metallurgy and Materials Science, College of Engineering and Physical Sciences, University of Birmingham, Edgbaston, Birmingham, B15 2SE, UK

^2^Nottingham Trent University, School of Science and Technology, Nottingham, UK

^3^Periodontal Research Group, Dentistry, School of Health Sciences, College of Medicine and Health, University of Birmingham, Edgbaston, Birmingham, B5 7EG, UK

^4^Birmingham Dental Hospital, Birmingham Community Health NHS Foundation Trust, Birmingham, United Kingdom

^5^ NIHR Birmingham Biomedical Research Centre, University of Birmingham, Birmingham, UK

^6^Neuroscience and Ophthalmology, Department of Inflammation and Ageing, School of Infection, Inflammation and Immunology, University of Birmingham, Edgbaston, Birmingham, B15 2TT, UK

^7^Centre for Trauma Sciences Research, University of Birmingham, Edgbaston, Birmingham, B15 2TT, UK

^8^University Hospitals Birmingham NHS Trust, Mindelsohn Way, Edgbaston, Birmingham, B15 2TT, UK

**Table S1.** Full search terms for all databases.

| **#** | **Query** |
| --- | --- |
| 1 | exp Antimicrobial Peptides/ |
| 2 | AMP*.mp. |
| 3 | exp Antimicrobial Cationic Peptides/ or exp Cathelicidins/ or host defence peptide.mp. |
| 4 | HDP.mp. |
| 5 | CHDP.mp. |
| 6 | exp beta-Defensins/ or exp alpha-Defensins/ or exp Defensins/ |
| 7 | exp Bacteriocins/ |
| 8 | exp Cecropins/ |
| 9 | antimicrobial cationic peptides/ or histatins/ or magainins/ or melitten/ or antimicrobial peptides/ |
| 10 | exp Nisin/ |
| 11 | exp Lactoferrin/ |
| 12 | protegrin.mp. |
| 13 | indolicidin.mp. |
| 14 | LL-37.mp. |
| 15 | hBD.mp. |
| 16 | human neutrophil peptide.mp. |
| 17 | HNP.mp. |
| 18 | Peptides, Cyclic/ |
| 19 | antibacterial peptide.mp. |
| 20 | antifungal peptide.mp. |
| 21 | antiviral peptide.mp. |
| 22 | 1 or 2 or 3 or 4 or 5 or 6 or 7 or 8 or 9 or 10 or 11 or 12 or 13 or 14 or 15 or 16 or 17 or 18 or 19 or 20 or 21 |
| 23 | Hydrogels/ |
| 24 | exp Nanofibers/ |
| 25 | Nanoparticles/ |
| 26 | composite*.mp. |
| 27 | polymer*.mp. |
| 28 | dressing*.mp. |
| 29 | exp Collagen/ |
| 30 | exp Chitosan/ |
| 31 | exp Alginates/ |
| 32 | exp Cellulose/ |
| 33 | exp Gelatin/ or gelatin*.mp. |
| 34 | exp Elastin/ |
| 35 | pullulan.mp. |
| 36 | Fibroins/ |
| 37 | exp Hyaluronic Acid/ |
| 38 | exp Polyethylene Glycols/ |
| 39 | polylactic acid.mp. |
| 40 | PEG.mp. |
| 41 | PLA.mp. |
| 42 | PLGA.mp. |
| 43 | PGA.mp. |
| 44 | polyglycolide.mp. |
| 45 | EPL.mp. |
| 46 | exp Polylysine/ |
| 47 | exp Lignin/ |
| 48 | PCL.mp. |
| 49 | polycaprolactone.mp. |
| 50 | PVA.mp. |
| 51 | exp Polyvinyl Alcohol/ |
| 52 | PEO.mp. |
| 53 | polyethylene oxide.mp. |
| 54 | 23 or 24 or 25 or 26 or 27 or 28 or 29 or 30 or 31 or 32 or 33 or 34 or 35 or 36 or 37 or 38 or 39 or 40 or 41 or 42 or 43 or 44 or 45 or 46 or 47 or 48 or 49 or 50 or 51 or 52 or 53 |
| 55 | cutaneous.mp. |
| 56 | dermal.mp. |
| 57 | Skin/ |
| 58 | lesion.mp. |
| 59 | wound.mp. |
| 60 | 55 or 56 or 57 |
| 61 | 58 or 59 |
| 62 | Infections/ |
| 63 | infected.mp. |
| 64 | 62 or 63 |
| 65 | 60 and 61 |
| 66 | 22 and 54 and 64 and 65 |
| 67 | limit 66 to (english language and yr="2013 -Current") |

**Table S2.** Details of Antimicrobial Peptide Sequences Used in the Included Studies. Brackets indicate sequence determined via literature review. NMeV = N-methylvaline; Sar = Sarcosine.

| **Study** | **Antimicrobial Peptide Sequence** |
| --- | --- |
| Fan et al., 2024 | KGRT |
| Heunis et al., 2013 | (MSTKDFNLDLVSVSKKDSGASPRITSISLCTPGCKTGALMGCNMKTATCHCSIHVSK)^1^ |
| Huan et al., 2022 | (MSTKDFNLDLVSVSKKDSGASPRITSISLCTPGCKTGALMGCNMKTATCNCSIHVSK)^1^ |
| Hussain et al., 2021 | KKFEFEFEFEKK |
| Lei et al., 2020 | KRWWKWWRR dimer |
| Wang et al., 2023 | KKLRLKIAFK |
| Wang-Guixi et al., 2024 | (SSRRKPCKGWLCKLKLRGGYTLIGSATNLNRPTYVRA)^2^ |
| Wang et al., 2024 | (KFFRKLKKSVKKRAKEFFKKPRVIGVSIPF)^3^ |
| Li et al., 2023 | GIIKKIIKKI |
| Lin et al., 2023 | YSLQM GATAI KQVKK LFKKK GG |
| Ni et al., 2024 | (GIGAVLKVLTTGLPALISWIKRKRQQ)^3,4^ |
| Wang Guan yi et al., 2024 | REERWF |
| Zhou et al., 2023 | (T-V-P-Sar-NMeV, T-V-oxoP-Sar-NMeV)^5^ |

**References**

1. The UniProt C. UniProt: the Universal Protein Knowledgebase in 2025. Nucleic Acids Research. 2025;53(D1):D609-D17.

2. Shi Y, Li C, Wang M, Chen Z, Luo Y, Xia X-s, et al. Cathelicidin-DM is an Antimicrobial Peptide from Duttaphrynus melanostictus and Has Wound-Healing Therapeutic Potential. ACS Omega. 2020;5(16):9301-10.

3. Salnikov E, Adélaïde M, Ramos-Martín F, Saad A, Schauer J, Cremanns M, et al. Cathelicidin-BF: A Potent Antimicrobial Peptide Leveraging Charge and Phospholipid Recruitment against Multidrug-Resistant Clinical Bacterial Isolates. Journal of the American Chemical Society. 2025;147(13):11199-215.

4. Compound Summary for Melittin [Internet]. National Center for Biotechnology Information. [cited 30 Jul 2025]. Available from: <https://pubchem.ncbi.nlm.nih.gov/compound/Melittin.Shi>

5. Qureshi KA, Bholay AD, Rai PK, Mohammed HA, Khan RA, Azam F, et al. Isolation, characterization, anti-MRSA evaluation, and in-silico multi-target anti-microbial validations of actinomycin X(2) and actinomycin D produced by novel Streptomyces smyrnaeus UKAQ_23. Sci Rep. 2021;11(1):14539.

**Table S3: Wound Characteristics.** FT = full thickness; CFU = colony forming units; MRSA: methicillin resistant *S. aureus*

| **Study** | **Wound Creation** | **Wound Infection** | **Duration of Exposure (Days)** |
| --- | --- | --- | --- |
| Fan et al., 2024 ^23^ | Circular FT wound, 10 mm diameter on dorsum | *S. aureus* ATCC 6538, 50 µL, 1 × 10^6^ CFU/ml | 14 |
| Heunis et al., 2013 ^32^ | Circular FT biopsy punch wound, 5 mm diameter on dorsum | *S. aureus* Xen 36, 10 μL, 1 × 10^8^ CFU/ml | 7 |
| Huang et al., 2022 ^20^ | Circular FT wound, 10 mm diameter on dorsum | *S. aureus* CVCC 546, 100 µL, 1 × 10^6^ CFU/100 μL | 14 |
| Hussain et al., 2021 ^28^ | Circular FT biopsy punch wound, 7 mm diameter on dorsum | *S. aureus*, 40 μL, 1 × 10^8^ CFU/mL | 14 |
| Lei et al., 2020 ^26^ | Oval FT biopsy punch wound, 10 mm long axis through panniculus carnosus | *S. aureus* ATCC 6538, 1 × 10^7^ CFU/mL | 10 |
| Wang et al., 2023 ^27^ | Oval FT biopsy punch wound, 8 mm x 6 mm, across panniculus carnosus | *S. aureus* ATCC 6538, 1 × 10^7^CFU/mL | 10 |
| Wang Guixi et al., 2024 ^21^ | Circular FT wound, 6 mm diameter on dorsum, cut with scissors | *S. aureus* ATCC25923, 20 μL 1 × 10^8^ CFU/mL | 12 |
| Wang Y et al., 2024 ^24^ | Circular FT wound, 6 mm diameter on dorsum | *S. aureus,* 1 × 10^6^ CFU/mL | 12 |
| Li et al., 2023 ^26^ | Circular FT wound, 10 mm diameter, cut with ophthalmic scissors on dorsolateral part | MRSA ATCC 43300, 10 μL, 1 × 10^8^ CFU/mL | 14 |
| Lin et al., 2023 ^30^ | Circular FT biopsy punch wound, 10 mm diameter on dorsum | MRSA ATCC 33591, 40 μL, 1 × 10^6^ CFU/mL | 10 |
| Ni et al., 2024 ^22^ | Circular wound, 15 mm diameter on upper dorsum | *S. aureus,* 200 µL, 1 × 10^6^ CFU/mL | 14 |
| Wang Guanyi et al., 2024 ^25^ | Square wound, 15 mm side length on dorsum | *S. aureus* | 15 |
| Zhou et al., 2023 ^31^ | Circular FT wound, 10 mm diameter on dorsum | *S. aureus,* 10 μL, 1 × 10^8^ CFU/mL | 21 |

**
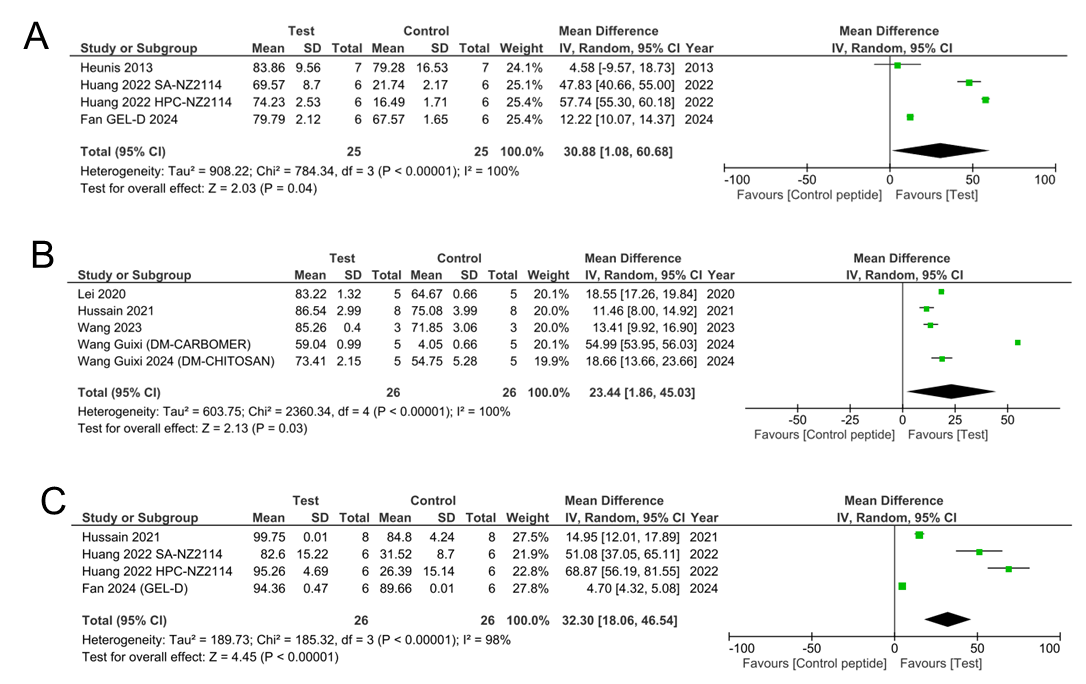
**

**FIGURE S1:** Meta-analysis of pooled data for wound healing rates in mice over time. Forest plots to show wound healing rates at (**A**) 7, (**B**) 10, and (**C**) 14 days after injury and treatment.


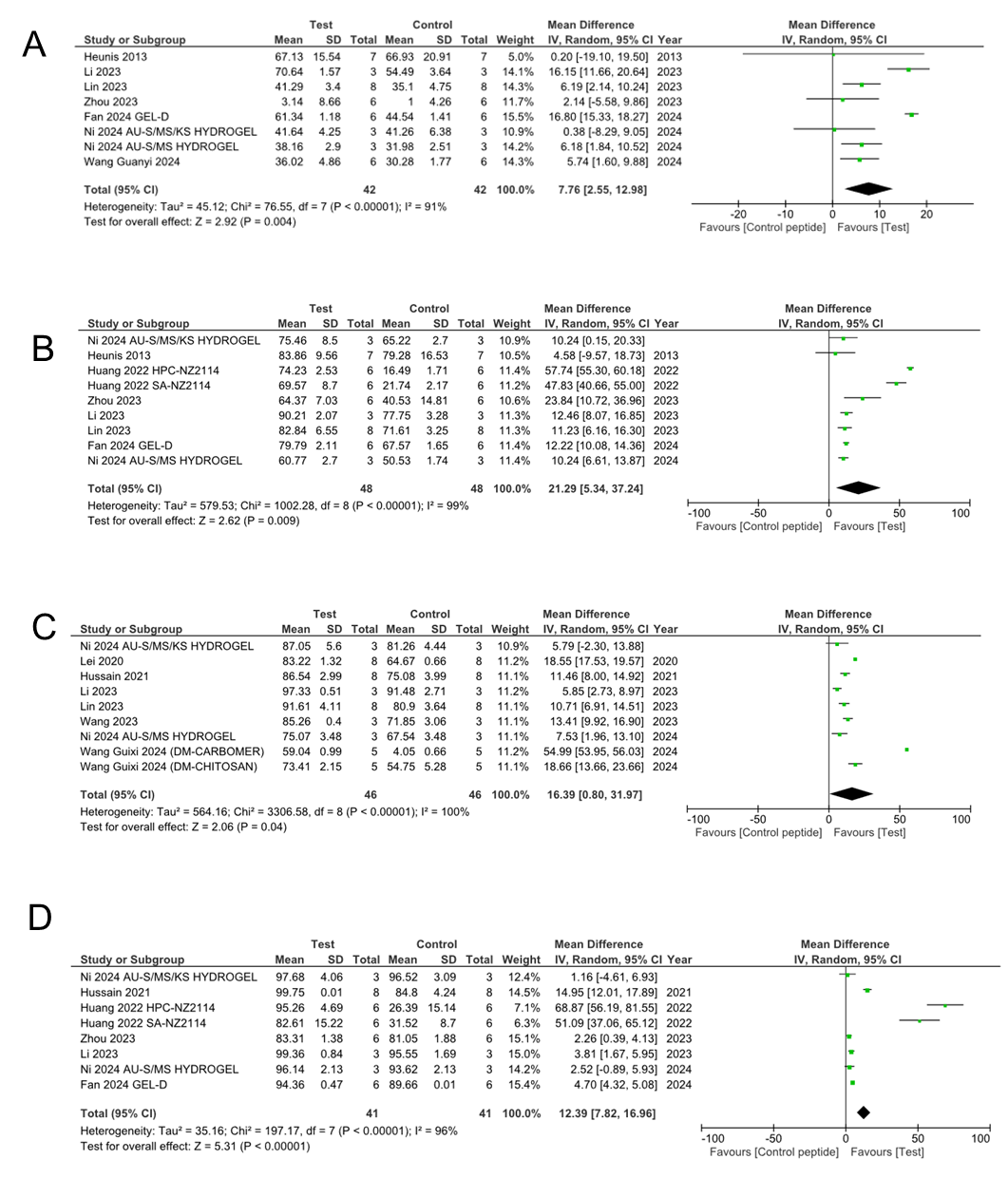


**FIGURE S2: Meta-analysis of pooled data for wound healing rates in rats and mice combined over time.** Meta-analysis shows wound healing rates at (**A**) 3, (**B**) 7, (**C**) 10 and (**D**) 14 days after injury and treatment.
